# The National Pension Fund

**Published:** 1888-03-17

**Authors:** 


### The National Pension Fund for Nurses.

The National Pension Fund, so long talked of, the object of so much interest and speculation among all who could hope to profit by it, is to-day an accomplished fact. Its memorandum and articles of association lie before us, and all who choose may learn exactly what it proposes to do, and how it means to do it. Copies of this publication may be had from the Secretary, 30, Old Jewry, on payment of one shilling per copy.

The general object of the Pension Fund is, indeed, obvious from its title, but many of those who are interested in it do not seem to have grasped the fact that the Pension Fund Society is an insurance company which issues policies, making provision for illness, permanent or temporary, accident, and old age. It is not, in the narrow sense of the term, a charitable association; that is, it does not say to any nurse, "Come here when you are ill or disabled from any cause, and the Fund will provide for you without asking any question as to your right to the title you bear, the work on which you have been employed, your opportunities for saving, and your claim upon its money." Its provisions are at once broad and just; while its purpose is to confer annuities on nurses of all descriptions, and officials of hospitals and other kindred institutions, and all institutions whose objects embrace, directly or indirectly, the relief or assistance of the sick, the injured, or the distressed, it is determined that those who profit by it shall show a due regard to their own position by setting aside a fair portion of their savings every year.

It is already known that the neccessary guarantee fund of £20,000 has been received, and that the interest of this sum will go to the income of the Fund. Around this golden nest-egg the contributions of the nurses will accumulate and increase by contact. The sum thus formed will be invested in ways common to insurance companies, and will be used to purchase and deal in reversionary interests, and, when desired, to acquire or extinguish by purchase or surrender any annuity policy issued by the Society. That is, to state the matter more plainly, an insurance policy issued by the Society which has united to form the Pension Fund is a piece of property belonging to the nurse in whose favour it is drawn out, whose value increases with every premium she pays at a fixed and ascertainable rate, and which she can at any time sell to the Fund, *i.e.*, she can withdraw the savings she has invested, with certain bonus additions. It has been said, "Young nurses won't invest any part of their income in the Pension Fund, for they don't know that they will ever become dependent on it. They may marry; they may inherit money; many accidents may happen which will make them cease paying their premiums, and then what they have already paid will be wasted."

Not at all. The nurse who marries can sell her policy to buy her trousseau; she who inherits money can, if she choose, give the proceeds of it to the hospital or institution she has been working in; she who needs an immediate sum for herself or her kindred more than she expects to need a pension in old age, has in it a piece of marketable property, which she can sell to the Fund at a price directly proportionate to the amount she has paid in premiums.

Besides this matter of granting annuities, the Society purposes creating special funds, including a sickness relief fund, an institution fund, and a bonus fund, which will give special advantages to those policyholders who are eligible for them. By these means the minimum payment at a certain age of £15, £22, or £30, which a particular policy promises, may be increased to something very much greater. Friends and ex-employers who are interested in a particular institution and its nurses, or in a particular nurse, can thus invest money on their behalf, sure that they will receive it with interest.

So much for the objects of the Society which has created the National Pension Fund; now for a word as to the members of it. It should be generally understood that these are not persons who have any thought of making money out of it. It is distinctly set forth that no portion of the income and property of the Society shall be paid by way of dividend or bonus, or in any way, either directly or indirectly, to the members of the Society, and no annuity or policy shall be granted to any member. On the contrary, the members of the Society are to give to it, not to receive from it. They are divided into two classes—first, individuals who pay an annual subscription of £2 2*s.*, or a single payment of £25; secondly, the representatives of any hospital, corporation, or institution which gives an annual subscription of £5 5*s.*, or a single payment of not less than £50. This regulation places above suspicion the motives of the members of the Society, who, it is seen, forfeit all claim to profit by the Fund by the fact of joining it. No self-seekers will therefore be found on its Council, and as this will consist mostly of laymen—"outsiders," as some people put it, meaning others than nurses—there will be no chance of personal prejudice and individual notions coming in to invalidate the genuine and just claims of those who seek to participate in the benefits of the Pension Fund.

The Council—on whom will fall the responsibility of managing the Fund and adjudicating on the claims of would-be annuitants—will consist of a president, vice-presidents (who shall not number more than four), a treasurer, and not more than twenty members of the Society, appointed by a majority of the subscribers to the memorandum of association. After the 1st of January, 1890, by which time the 1,000 policies required by the donors of the guarantee fund must be granted, the Council will have eight other persons added to it, who will represent the annuitants and policy-holders of the Society.

The duties of the Council will be, as stated above, to elect applicants for pensions proposed by members of the Society, and to invest and manage the funds of the Society, fixing the amount of premiums for annuities and sickness policies, and the amount given as sickness allowances, according as the funds of the Society may justify. They must also formulate and circulate among the annuitants and policyholders of the Society regulations regarding the business to be undertaken by their representatives on the Council, and to enable these annuitants to hold meetings for the election of their representatives. It is enacted that any representative member who does not conform to this rule, who in any way tries to keep the annuitants in the dark, shall thereby be disqualified for membership in the Council.

Such, in brief, are the regulations of the National Pension Fund—regulations which must prove conclusively that it is founded by subscribers and carried on by gentlemen who have no thought but that of benefiting the nurses and others having the right to participate in it, and who are well able to carry out their philanthropic plans. All who are wise will join this Fund if they can only afford to enter for a small pension, even for one so small as £10 per annum at fifty years of age. The reason we give this advice to nurses is that the founders have shown the genuine desire that animates them to help nurses to help themselves to independence and comfort by raising £26,000 before issuing any prospectus or accepting a single premium. Those who have done this much spontaneously for nurses are entitled to their confidence individually and collectively. The less a nurse can do for herself the more she should determine to join this Fund at once, because those who have shown such a tender regard for all nurses may be trusted to take steps to secure that the Bonus Fund shall be ample to supplement the savings of every nurse who does her best to help herself.

